# Anticipating health and care trajectories from routinely collected social care records

**DOI:** 10.1038/s44401-026-00139-3

**Published:** 2026-08-29

**Authors:** Xiao Gu, Rebecca Nourse, Lei Lu, Emily Smout, Karen Fuller, Fiona Havord, Victoria Baran, Adam Briggs, Lei Clifton, Andrew Farmer, David A. Clifton

**Affiliations:** 1 Department of Engineering Science, University of Oxford, Oxford, UK; 2 Nuffield Department of Primary Care Health Sciences, University of Oxford, Oxford, UK; 3 School of Life Course and Population Sciences, King’s College London, London, UK; 4 Oxfordshire County Council, Oxford, UK; 5 University of Southampton, Southampton, UK

**Keywords:** Computational biology and bioinformatics, Health care, Mathematics and computing, Medical research

## Abstract

Predictive modelling in healthcare has advanced rapidly, yet social care systems, despite their central role in supporting vulnerable populations, remain underexplored in this domain. In this study, we apply machine learning to a large, pseudonymised dataset of social care records from 27,590 adults in Oxfordshire, encompassing around 90% of individuals receiving care in the region. We developed models to predict three outcomes of interest: future care plan needs, hospital admissions, and all-cause mortality, evaluated across multiple prediction horizons. Our results show that hospital admission and mortality can be predicted with meaningful discriminative performance (AUROC up to 0.893), while care plan needs are more variable and harder to predict. Post-hoc exploratory analyses revealed distinct risk signatures across outcomes, and temporal evaluation showed that care needs often emerge earlier than clinical deterioration. These findings demonstrate the feasibility of social care-based risk modelling and suggest new opportunities to improve anticipatory care planning, health management, and resource allocation using routinely collected non-clinical data.

## Introduction

The growing demand for adult health and social care in the United Kingdom, driven by population ageing, rising multimorbidity and disability, and wider social determinants such as poverty, poses major challenges to service delivery and sustainability^1^. These pressures are compounded by workforce shortages and limited financial resources across both health and social care systems^2–4^. At the same time, current NHS policy explicitly frames the coming decade as an opportunity to shift care away from hospital-centric models towards community- and home-based support, as part of its long-term, integrated care strategy^5,6^. Within this context, policy and research increasingly emphasize the importance of predictive analytics to enable care that is more proactive, preventive, and integrated^5,7^. Social care plays a central role in supporting vulnerable populations, often complementing and, at times, offering a viable alternative to traditional health care services. As patterns of disease evolve and new treatments become available, more individuals can safely receive care within their communities, making effective planning and coordination between social and health care services increasingly essential.

To date, most predictive models rely on clinical datasets, such as hospital and primary care records, to forecast health-related outcomes, *e.g*., predicting adverse outcomes^8,9^ and optimising treatment pathways^10^. Despite such extensive use of health care data for predictive modelling and resource planning, social care data remain comparatively underused. While clinical datasets capture detailed clinical information, they do not generally include functional, behavioural, and social factors that shape care needs^11,12^. In England, local authority social care records, routinely collected through statutory assessments, reviews, and service delivery, provide complementary information about individuals’ function, mobility, independence, and engagement with support services^3^. Many of these characteristics are specific to social care and may provide earlier indicators of health-related outcomes. Using these data offers an opportunity to develop more holistic and anticipatory approaches to care that improve both individual outcomes and system efficiency.

In this study, we explore how local authority adult social care records can be used to develop predictive models for three outcomes: initiation of a care plan, hospitalisation, and mortality. Leveraging a comprehensive dataset comprising pseudonymised social care records for around 28,000 adults in Oxfordshire, we applied a landmark-based dynamic prediction framework in which risk estimates are repeatedly generated at predefined time points, using all information available up to each prediction date. This approach enables evaluation of predictive performance across multiple prediction horizons, capturing both short- and long-term risks. We further conduct exploratory analyses of feature importance, risk trajectories, and cross-outcome relationships to better understand how social and health-related risks evolve over time. Together, these analyses provide new empirical evidence on the predictive value of social care data, highlighting opportunities to inform data-driven approaches to care in line with current policy priorities in England.

## Results

### Landmark prediction framework on longitudinal social care records

**Fig. 1:**
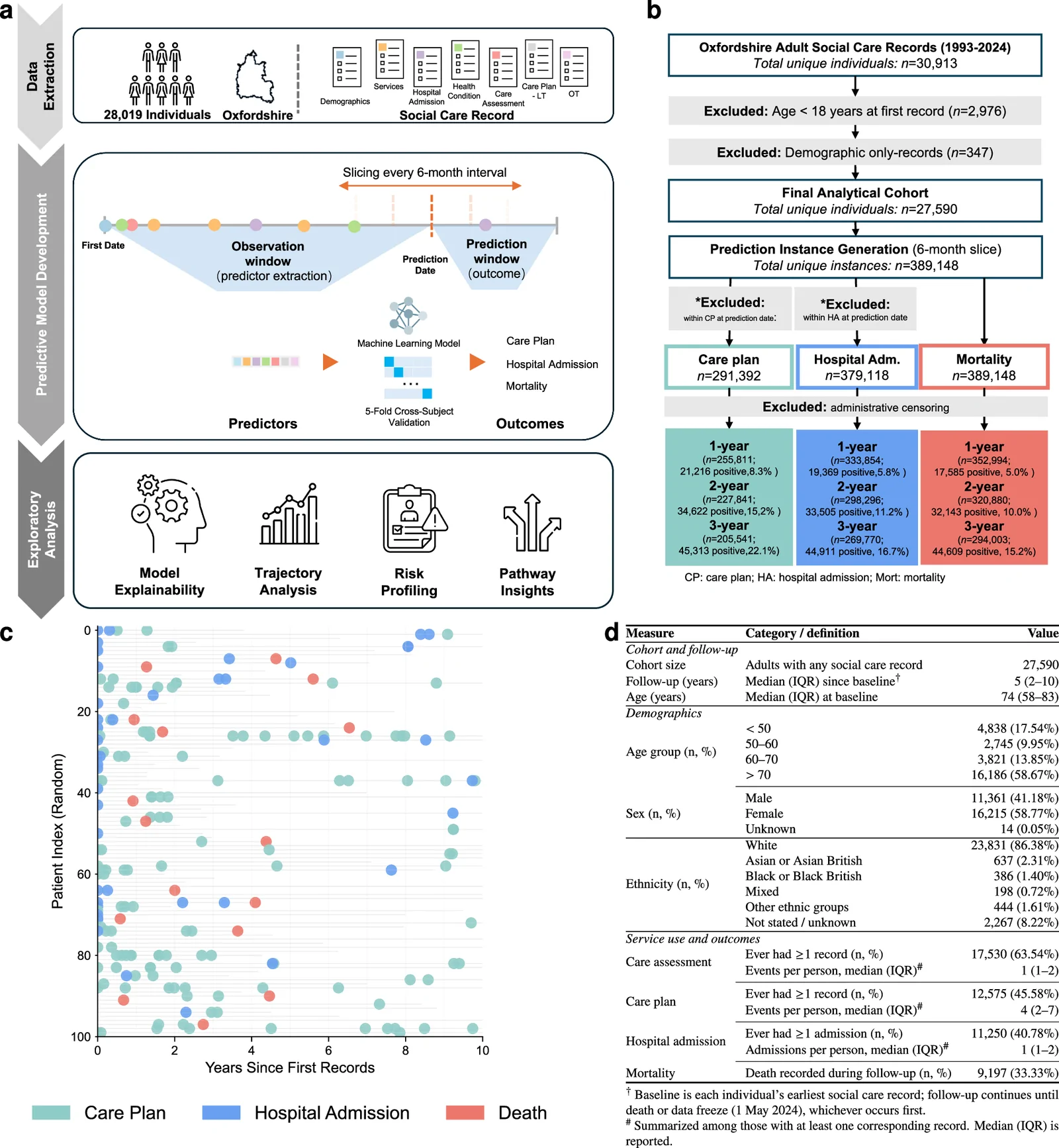
Overview of the Oxfordshire adult social care dataset and study design. **a** Overall study framework illustrating the three main stages: data extraction from routinely collected social care records, predictive model development using sequential 6-month observation and prediction windows, and exploratory analyses including model explainability, trajectory analysis, and risk profiling. **b** Flow diagram of cohort selection and prediction instance generation. **c** Representative patient-level trajectories for 100 randomly selected individuals, showing the timing of major events from each person’s first recorded contact. Green, blue, and red markers represent care-plan initiation, hospital admission, and death, respectively. **d** Baseline demographic and social/health-care characteristics of the analytical cohort.

We initially analysed pseudonymised longitudinal social care records from 30,913 adults in Oxfordshire, UK, spanning 1993–2024. The dataset was extracted and provided by Oxfordshire County Council, with 1 May 2024 marking the administrative end of follow-up. It comprised detailed structured records across seven domains, including demographics, service utilisation, care assessments, care plans, occupational therapy (OT) equipment and reablement, self-reported health conditions, and hospital admissions, as illustrated in Fig. 1a. An example of the raw data schema is provided in Supplementary Table S1. The study was conducted under a data-sharing agreement between Oxfordshire County Council and the University of Oxford, with pseudonymised data analysed on secure University of Oxford servers. Full details of data governance, pseudonymisation, and approvals are provided in the Methods.

After excluding individuals younger than 18 years at their first recorded entry and those with demographic-only records, the final analytical cohort comprised 27,590 adults. The cohort had a median age of 74 years (IQR: 58–83, calculated at each individual’s earliest social care record), 58.5% were female, and the median follow-up duration was 5 years (IQR: 2–10). During follow-up, 45.6% initiated at least one care plan, 40.8% had at least one hospital admission recorded in the social care data, and 33.3% died.

We focused on three outcomes that reflect different aspects of health and care trajectories: initiation of a new long-term care plan, hospital admission, and all-cause mortality. Representative longitudinal trajectories are shown in Fig. 1c, where green, blue, and red markers illustrate the heterogeneous timing of care-plan, hospital-admission, and death events, respectively.

A key challenge in analysing these records is that individuals do not share a single common baseline assessment. Instead, information accumulates irregularly as people come into contact with social care services over time. We therefore used a landmark prediction approach^9,13^, in which each individual’s record was reviewed at successive 6-month intervals. At each interval, information recorded up to that point was used to estimate the risk of care-plan initiation, hospital admission, or death within the following 1, 2, or 3 years. This allowed each individual to contribute multiple prediction instances over time, reflecting how their available information changed as their social care record developed.

After applying prediction-instance eligibility criteria, the landmark prediction process yielded 291,392 care-plan prediction instances, 379,118 hospital-admission prediction instances, and 389,148 mortality prediction instances (Fig. 1b). These repeated prediction instances formed the analysis set for the 1-, 2-, and 3-year prediction horizons reported below. Detailed descriptions of outcome definitions, predictor construction, censoring rules, model development, calibration, and validation are provided in the Methods and Supplementary Section S1.

### Social care records enable risk prediction across outcomes and horizons

As a proof-of-concept study, we first assessed the overall predictive performance of routinely collected social care records across three clinically and operationally relevant outcomes: care-plan initiation, hospital admission, and all-cause mortality. Predictions were evaluated across 1-, 2-, and 3-year horizons.

To assess performance across models with different levels of complexity, we compared logistic regression, random forest, and XGBoost under the same repeated prediction framework. Models were evaluated using subject-wise five-fold cross-validation, with the area under the receiver operating characteristic curve (AUROC) as the primary discrimination metric and calibration assessed using calibration curves and Brier scores; full experimental protocols and additional metrics are reported in the Methods and Supplementary Section S1 and Table S3.

**Fig. 2:**
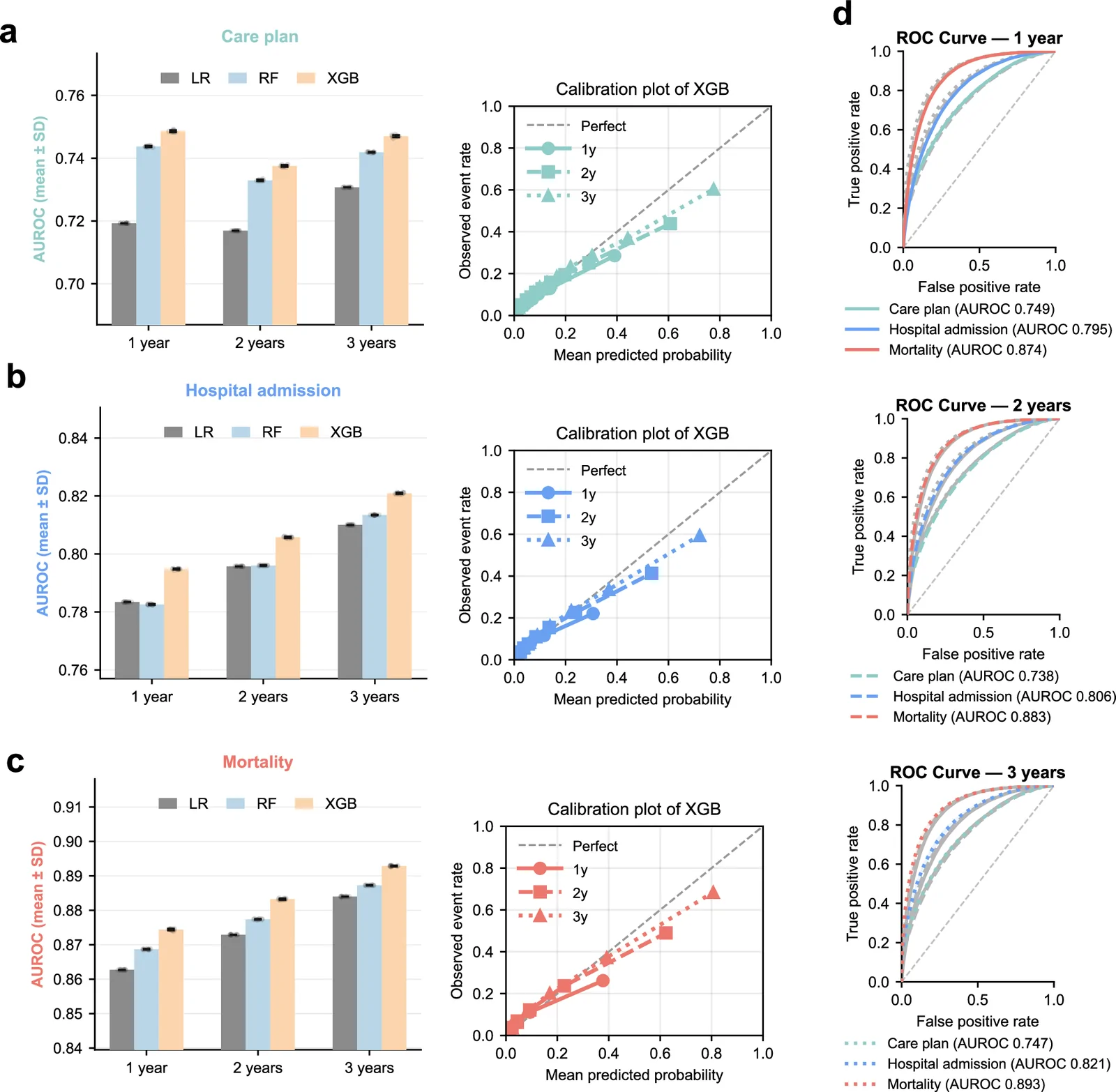
Risk prediction performance across outcomes and time horizons using social care records. Discrimination and calibration analyses for predicting care plan initiation (**a**), hospital admission (**b**), and all-cause mortality (**c**) at 1-, 2-, and 3-year horizons. Left panels present mean AUROC ± standard deviation across cross-validation splits for logistic regression (LR), random forest (RF), and XGBoost (XGB) models. Individual dots represent results from 10 random seeds. Middle panels show calibration curves for XGBoost, comparing mean predicted probabilities with observed event rates at each horizon. **d** Receiver operating characteristic (ROC) curves for the three outcomes within each prediction horizon.

The main predictive performance results across outcomes and prediction horizons are summarised in Fig. 2a–c. Across all outcomes and horizons, the models achieved meaningful discrimination, indicating that social care records contain informative signals for future health and care risk. Among the evaluated models, XGBoost generally achieved the strongest discrimination across outcomes and horizons compared with logistic regression and random forest models. We therefore used XGBoost as the primary model for subsequent calibration, feature-attribution, and trajectory analyses. Calibration curves and receiver operating characteristic curves are shown in Fig. 2a–c right panels and Fig. 2d, respectively, and are described in more detail below.

Predictive performance varied by outcome. Mortality was consistently the most discriminable endpoint, followed by hospital admission, while care-plan initiation was comparatively harder to predict (Fig. 2a–c). Using XGBoost, AUROC ranged from 0.874 to 0.893 for mortality, from 0.795 to 0.821 for hospital admission, and from 0.738 to 0.749 for care-plan initiation across the three prediction horizons.

The comparatively lower discrimination for care-plan initiation may reflect its nature as a service-response outcome. Unlike mortality or hospital admission, which are more directly linked to accumulated health burden and acute deterioration, the start of a care plan may also depend on wider social and contextual factors that are not fully captured in routine records, including informal care availability, living arrangements, housing conditions, financial circumstances, individual preferences, and local service capacity. Likewise, because NHS data linkage was not available for hospital admission, performance should therefore be interpreted against the recorded hospital-admission endpoint available in the social care records, rather than as validation against complete NHS hospital-admission records.

Performance also varied across prediction horizons. For hospital admission and mortality, discrimination increased with longer prediction windows, suggesting that individuals who experienced these events became more separable from those who did not over extended follow-up. For care-plan initiation, performance was broadly similar across horizons, indicating that short- and longer-term care-plan needs may be less distinctly separable using the available routinely collected variables.

Receiver operating characteristic curves further confirmed the outcome-specific pattern of discrimination, with the clearest separation for mortality, followed by hospital admission and care-plan initiation (Fig. 2d).

Calibration analysis of the XGBoost models showed good overall agreement between predicted probabilities and observed event rates across outcomes and time horizons, with modest deviations mainly at higher predicted-risk levels (Fig. 2a–c, right panels). Together, these findings indicate that routinely collected social care records can support repeated risk prediction across multiple outcomes, while also highlighting important differences in predictability between future care needs, hospital admission, and mortality.

### Interpretability analysis highlights outcome-specific features linked to care pathways

**Fig. 3:**
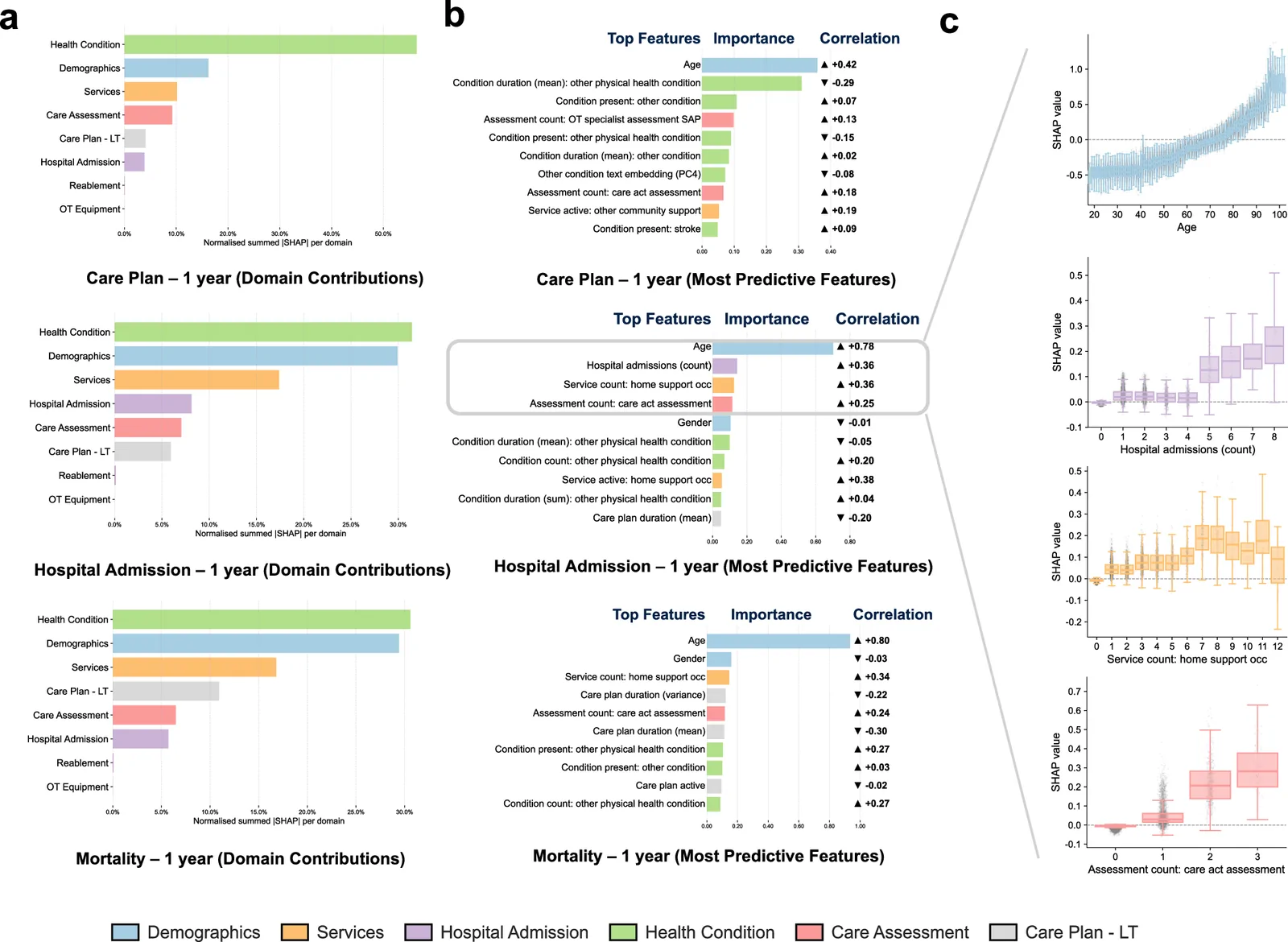
Predictive feature contributions across outcomes at the 1-year horizon. **a** Domain-level contributions to model predictions for care plan initiation, hospital admission, and mortality, computed as the normalized sum of absolute SHAP values within each feature domain. **b** Top predictive features for each outcome, ranked by mean absolute SHAP value, with accompanying direction of association (positive or negative correlation with risk). **c** SHAP dependence plots show how predicted risk changes as individual feature values increase. Each point represents one prediction instance, with higher values on the vertical axis indicating greater contribution to predicted risk.

To further interpret the drivers of model predictions, we examined feature importance at both the domain and individual feature levels for the 1-year horizon, focusing on the best-performing models. Feature attributions were derived using SHAP (SHapley Additive exPlanations) and aggregated to highlight interpretable patterns across outcomes (Fig. 3), with the left panel Fig. 3a reporting domain-level aggregated contributions, whereas the middle panel Fig. 3b shows individual feature-level contributions.

Figure 3 a shows the proportion of total absolute SHAP importance attributable to each data domain. At the domain level, features capturing health conditions accounted for the largest share of predictive importance across all outcomes, highlighting the dominating role of self-reported health conditions in risk stratification. This dominance likely reflects, at least in part, the greater breadth and granularity of variables available within the health conditions domain (refer to Table S2). Notably, for care plan initiation (1-year), the health condition domain dominated more strongly relative to other domains than observed for hospital admission or mortality. This pattern suggests that care plan initiation may be more directly driven by assessed clinical and functional needs, rather than by downstream adverse outcomes such as hospitalisation or death.

Across outcomes, service-related domains consistently ranked behind health conditions and demographics, typically as the third most influential domain (Fig. 3a). This suggests that prior service utilisation, which reflects patterns of existing service engagement, carries meaningful predictive information for health-related outcomes.

Figure 3 b presents feature-level contributions, with age emerging as the most influential predictor across all outcomes. SHAP dependence plots, which illustrate the relationship between a feature’s value and its contribution to predicted risk, revealed a smooth, monotonic increase in risk with advancing age (Fig. 3c, top). This indicates that age contributes cumulatively rather than through a sharp threshold effect, consistent with its role as a proxy for frailty, multimorbidity, and declining physiological reserve.

Several features ranked among the most predictive for non-mortality outcomes (Fig. 3b, highlighted rows). Recurrent hospital admissions were strongly associated with increased 1-year risk for both care plan initiation and subsequent hospital admission, with SHAP values increasing sharply after multiple admissions (Fig. 3c, second panel). Similarly, higher frequencies of home support services and repeated care assessments were linked to elevated predicted risk (Fig. 3c, lower panels). Importantly, these associations do not imply that service use itself increases risk; rather, they likely reflect escalating care needs, instability, or unmet support requirements that prompt repeated contact with health and social care services.

For mortality prediction, service-related features (service count: home support occ) also contributed as the third most predictive feature (Fig. 3b, bottom), indicating the mortality risk relationship with the patterns of service engagement. Notably, measures related to care plan duration were among the most influential predictors for mortality at the individual feature level, exhibiting a consistent negative association with predicted risk. Both longer mean care plan duration and greater variability in duration were associated with lower mortality risk. Rather than indicating a protective effect of prolonged care plans, this pattern likely reflects care stability and continuity: long-standing and actively managed care plans may correspond to well-established support arrangements, whereas shorter or newly initiated care plans may signal recent deterioration, care transitions, or end-of-life processes associated with elevated mortality risk.

We additionally performed a fold-internal feature-selection sensitivity analysis for the 1-year XGBoost models, with details provided in Supplementary Fig. S2.

### Multi-outcome risk stratification and temporal analysis

To examine whether the fixed-horizon risk scores reflected meaningful longitudinal care trajectories, in an exploratory manner, we performed post-hoc analyses using the calibrated predicted probabilities assigned to each eligible prediction instance, following similar risk-stratification analyses in previous work^14–17^. Within each cross-validation split, model training and calibration were first completed using the training data only (1-year horizon prediction models). The trained models were then applied to eligible instances in the held-out set to generate calibrated risk scores, including instances that were subsequently censored or experienced competing events. These scores were used descriptively for survival, competing-risk, and temporal trajectory analyses. These post hoc analyses were not used for model training, tuning, threshold selection, or optimisation, but were intended to assess whether predicted risks stratified subsequent time-to-event patterns.

**Fig. 4:**
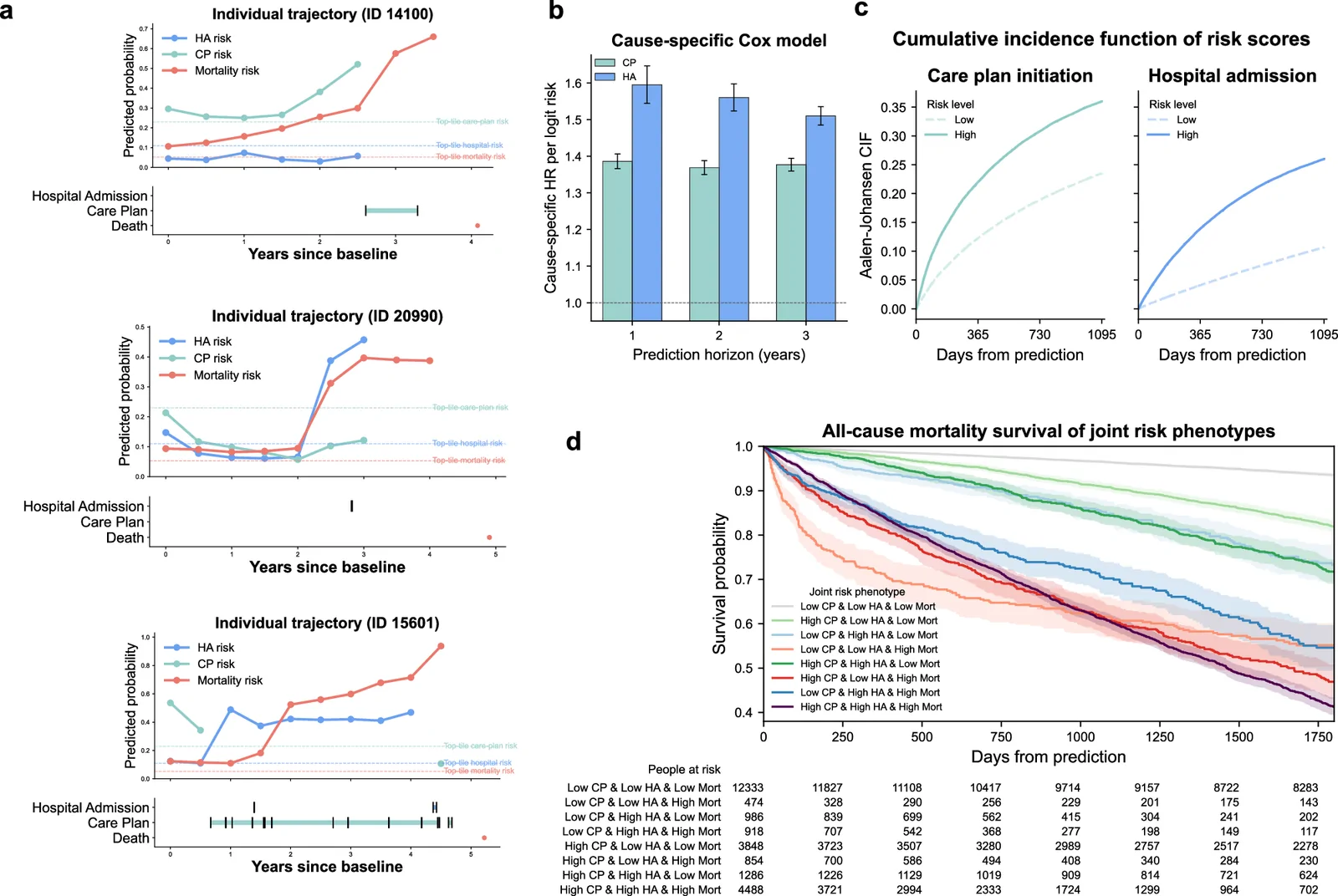
Individual risk trajectories and population-level risk stratification across social care outcomes. **a** Example individual trajectories showing how predicted risks changed over time for hospital admission (HA), care-plan initiation (CP), and all-cause mortality. The upper part of each example shows the model-predicted probabilities at different time points, and the lower part shows the observed events for the same person, including hospital admissions, care-plan episodes, and death. Dashed horizontal lines show the thresholds used to define high-risk status for each outcome. **b** Cause-specific Cox analysis showing whether higher model-predicted risk was associated with a higher rate of the target event. Separate estimates are shown for CP and HA at the 1-, 2-, and 3-year prediction horizons. Error bars show 95% confidence intervals, and the dashed horizontal line marks no association. **c** Aalen--Johansen cumulative incidence curves for CP and HA. These curves show the observed probability of each target event over time in high-risk and lower-risk groups, while accounting for death as a competing event that can occur before CP or HA. **d** Kaplan--Meier survival curves for groups defined by the combined CP, HA, and mortality risk predictions at baseline. For each outcome, high risk was defined as the top 20% of held-out predicted risks, and these high/low indicators were combined to form eight joint risk phenotypes. Shaded areas show 95% confidence intervals, and the table below shows the number of individuals still under observation over time. This analysis used all-cause death as the endpoint and was intended as a post-hoc survival stratification analysis.

First of all, to better understand the predicted risks, we present three representative trajectories showing distinct patterns of risk dynamics aligned with observed events (Fig. 4a). In one individual (top panel), predicted care plan risk increased gradually over time, followed by a later sharp rise in mortality risk, while hospital admission risk remained comparatively low throughout. In another example (middle panel), predicted risks across all three outcomes rose sharply within a short time window, closely aligned with observed hospital admission and death events. A third trajectory shows persistently elevated hospital admission risk accompanied by multiple hospital admission events, with mortality risk increasing more gradually rather than abruptly. Across these examples, predicted risks varied in both timing and magnitude across outcomes and were differently aligned with observed care events. These trajectories are presented as illustrative cases to demonstrate the diversity of individual-level risk dynamics captured by the model.

We further examined the two non-mortality outcomes, care-plan initiation and hospital admission, with death before the target endpoint handled as a competing event. To assess whether the calibrated fixed-horizon risk scores remained informative under this setting, we first fitted cause-specific Cox models using the calibrated predicted risk score as the exposure. Following the standard cause-specific hazard formulation, the target endpoint was treated as the event of interest and individuals who died before the target endpoint were censored at the time of death.

In the cause-specific hazard analysis, as shown in Fig. 4b, higher calibrated risk scores were consistently associated with higher target-event hazards across all prediction horizons. For care-plan initiation, the hazard ratio per one-unit increase in logit predicted risk was 1.39 (95% CI 1.37–1.41) at 1 year, 1.37 (95% CI 1.35–1.39) at 2 years, and 1.38 (95% CI 1.36–1.39) at 3 years. For hospital admission, the corresponding hazard ratios were 1.59 (95% CI 1.54–1.65), 1.56 (95% CI 1.52–1.60), and 1.51 (95% CI 1.49–1.54), respectively.

We also estimated Aalen–Johansen cumulative incidence functions (CIFs), as shown in Fig. 4c, explicitly encoding death before the target endpoint as the competing event. For each endpoint, individuals were stratified using the endpoint-specific calibrated predicted risk score, with the high-risk group defined as the top 20% of predicted risk and the lower-risk group defined as the remaining 80%. These analyses showed clear separation between high- and lower-risk groups when competing mortality was accounted for. By 3 years, the cumulative incidence of care-plan initiation was approximately 0.55 in the high-risk group compared with 0.31 in the lower-risk group. For hospital admission, the corresponding cumulative incidence was approximately 0.44 in the high-risk group compared with 0.13 in the lower-risk group.

After assessing the two non-mortality endpoints under a competing-risk framework, we examined whether combined risk profiles across care-plan initiation, hospital admission, and mortality were associated with subsequent survival. Individuals were stratified into joint multi-outcome risk phenotypes using their calibrated fixed-horizon predicted risks across the three outcomes. For each outcome, high risk was defined as the top 20% of the endpoint-specific predicted-risk distribution, and the resulting high/low indicators were combined to define eight joint phenotypes. We then used Kaplan–Meier curves to compare subsequent survival across these phenotypes, with all-cause death as the endpoint (Fig. 4d). This analysis was intended as a post-hoc survival stratification analysis, rather than as a competing-risk analysis for care-plan initiation or hospital admission.

The Kaplan–Meier analysis showed clear separation in subsequent survival across the eight joint risk phenotypes. Individuals with low predicted risk across all three outcomes had the most favourable survival trajectory, whereas those classified as high risk across all three outcomes had the poorest survival. Survival was also reduced in phenotypes involving high predicted mortality risk, even when predicted care-plan initiation and hospital-admission risks were not elevated. This suggests that the mortality model captured vulnerability signals that were not fully reflected in future care-transition or hospital-admission risk. Elevated care-plan and hospital-admission risks provided additional context, identifying groups with broader multi-domain vulnerability. Overall, these findings suggest that the fixed-horizon models captured related but non-identical dimensions of risk, and that their combined risk profiles may provide early warning signals for longer-term survival trajectories.

It should be noted that these findings should be interpreted as descriptive post-hoc analyses based on model-derived risk phenotypes. The phenotypes reflect predicted future-event risk among eligible individuals, rather than current care-plan or hospital-admission status at baseline. They should therefore not be interpreted as causal effects of care-plan or hospital-admission risk on mortality, or as formal competing-risk estimates for the non-mortality endpoints. As the study was conducted within a single local authority dataset, the generalisability of these patterns to other health and social care systems remains uncertain and requires external validation.

## Discussion

This study shows that routinely collected adult social care records can support prospective risk estimation for multiple outcomes that matter to both health service planning and clinical pathways: initiation of a care plan, hospital admission, and all-cause mortality. Using longitudinal records from around 28,000 adults in Oxfordshire, we developed and validated dynamic, landmark-based prediction models across 1-, 2-, and 3-year horizons, demonstrating meaningful and consistent discrimination across tasks.

Model performance differed by outcome in a pattern that is informative for interpretation and use. Mortality was the most predictable endpoint, hospital admission showed moderate-to-strong discrimination, and care plan initiation was comparatively harder to predict. This gradient likely reflects the nature of each endpoint: mortality and hospital admission are more directly driven by underlying health burden and accumulated vulnerability captured in the records, whereas care plan initiation will be more significantly influenced by professional judgement, eligibility, local practice, service availability, and individual preferences. Importantly, this does not reduce the value of care plan prediction; rather, it highlights that it represents a distinct decision context and should be interpreted as a risk of future service need, not as a proxy for imminent clinical deterioration. Future work may therefore focus on identifying additional predictors of care plan initiation, particularly variables capturing functional status, social support, and assessment context, to better represent this decision process and improve predictive performance.

This interpretation also clarifies how the present work relates to established clinical approaches for older adults, which often focus on frailty, multimorbidity, hospital admission, or holistic assessment after clinical need has already become apparent^18–20^. Our aim is not to replace clinical frailty assessment or multidisciplinary review, but to test whether routinely collected social-care records provide an earlier and complementary signal of vulnerability in the community^21^. These records may capture information that is not central in many hospital-facing workflows, including functional support needs, care intensity, package changes, and patterns of service use. We therefore view the models as an upstream social-care-based risk-stratification layer that could help identify people for review, rather than as an automated decision tool or a substitute for clinical assessment.

Our interpretability analyses further support this distinction. Age and health-condition indicators were consistently influential across tasks, consistent with established links to frailty and multimorbidity. In addition, markers of prior system contact, such as repeated care assessments, home-support service use, and prior hospital admissions, contributed substantially to near-term risk in the corresponding models. These associations should be interpreted as predictive signals rather than causal effects: higher recorded service use likely reflects greater underlying need, instability, or care transitions that bring individuals into contact with services.

A key policy-relevant observation is the clear and sustained separation in survival across the joint risk phenotypes. Individuals jointly classified as high risk for mortality, hospital admission, and care plan initiation experienced the poorest long-term survival, whereas those with uniformly low predicted risk maintained excellent survival throughout follow-up (Fig. 4d). Importantly, several mixed phenotypes also showed distinct trajectories: in particular, high care plan risk coupled with high mortality risk but low hospital admission risk was associated with worse survival than some groups with elevated hospital admission risk, suggesting a vulnerable subgroup whose decline may be managed largely outside hospital settings. Conversely, high care plan risk in the presence of low mortality risk was associated with comparatively favourable survival, consistent with care planning capturing support needs that are not synonymous with imminent deterioration. Together, these patterns indicate that combining outcomes can reveal clinically and service-relevant heterogeneity that is obscured when outcomes are considered in isolation, and may help inform anticipatory care planning and coordination across health and social care services.

Several limitations are important for interpretation and generalisability. First, the study used data from a single local authority, and performance may vary across regions with different service configurations and recording practices. Second, key determinants of need and outcomes, including social isolation, housing quality, financial circumstances, and informal care, were not captured in our dataset, and may confound both service use and observed endpoints.

Third, outcomes were derived from routinely recorded administrative data and may therefore be subject to measurement error, timing uncertainty, and incomplete ascertainment. This is particularly relevant for hospital admission. Although hospital admissions were available in the social care records, direct linkage to Hospital Episode Statistics, primary care records, or other NHS administrative datasets was not available. The hospital-admission endpoint should therefore be interpreted as recorded hospital admission within the available social care data, rather than complete NHS admission risk. We could not quantify how many emergency or elective admissions were not captured. Missed admissions may introduce label noise or systematic bias if recording completeness is related to social care contact, care intensity, or other patient characteristics. Future linkage with NHS hospital and primary care records is needed to quantify outcome capture, improve clinical contextualisation, and evaluate cross-sector care trajectories.

Future implementation should also address inequality explicitly. Frailty and care needs are socially patterned, with evidence linking household wealth and neighbourhood deprivation to frailty trajectories in England^22^. Future external validation should therefore report discrimination and calibration across demographic and socioeconomic groups, including age, sex, ethnicity, deprivation, geography, and disability where available. Beyond external validation, linked NHS records, coded frailty measures, and community clinical data could help test whether social-care signals remain complementary after adding diagnoses, prescribing, physiology, and validated frailty assessments. Passive digital-health sources, such as wearable or home-sensor data, should be considered only as future complementary inputs and would require appropriate evidence, governance, privacy protection, accessibility safeguards, human oversight, and non-digital alternatives.

In conclusion, routinely collected social-care records may provide an earlier and complementary signal of vulnerability that can support anticipatory review, resource planning, and coordination across health and social care. These models should be interpreted as decision-support tools for prioritisation, not as automated decision-making systems. Future work should prioritise external validation, prospective workflow evaluation, linkage with health records, and careful assessment of calibration, equity, and implementation capacity.

## Methods

### Data Sources and Cohort Description

This retrospective analysis leverages a comprehensive dataset of pseudonymised social care records from 30,913 adults in Oxfordshire, UK, spanning 1993–2024. The records encompass detailed structured records across seven domains, including demographics, service utilisation, care assessments, care plans, occupational therapy (OT) equipment and reablement, self-reported health conditions, and hospital admissions. Such rich longitudinal data enable a robust investigation into the trajectories of care needs, healthcare utilisation, and overall outcomes in this population.

This work was approved by Oxfordshire County Council’s Data Governance Board and conducted under a data-sharing agreement between Oxfordshire County Council and the University of Oxford. All data were pseudonymised prior to transfer and were effectively anonymised in the hands of the Oxford research team, who had no access to the re-identification key. The data were analysed on secure University of Oxford servers. In line with UK guidance on the secondary analysis of anonymised datasets, individual consent and research ethics committee approval were not required. As this was a retrospective exploratory analysis, no separate public study protocol was prepared, and the study was not prospectively registered.

We initially analysed records from 30,913 adults. Individuals were excluded if they were younger than 18 years at their first recorded entry or had demographic-only records. After applying cohort-level and prediction-instance eligibility criteria, the final analytical cohort comprised 27,590 adults who contributed at least one valid prediction instance, as shown in Fig. 1b.

### Outcome measures

We defined three primary outcomes of interest (Fig. 1a,c): *(1) Care Plan Initiation*, representing the start of a new long-term care plan following the prediction date, defined by the first effective start date not overlapping with any prior plan. This outcome serves as a marker for the need to adjust or implement care strategies in response to evolving patient requirements. *(2) Hospital Admission*, defined as the occurrence of at least one inpatient episode within the prediction horizon, based on admission dates recorded in the available social care records. This endpoint therefore represents hospital admissions recorded within the social care dataset, rather than complete ascertainment of all NHS emergency or elective admissions. *(3) All-Cause Mortality*, determined from the recorded year of death in the demographic record.

Collectively, these outcomes provide a comprehensive framework to assess and predict the future trajectory of social care needs, healthcare utilisation, and survival.

### Prediction instance construction

In this retrospective study, there was no single predefined “baseline” or “assessment” date for each individual, due to the longitudinal and irregular nature of the social care records. To structure these records for prediction, we adopted a landmark-based dynamic prediction framework, in which predictions were repeatedly generated at predefined time points using all information available up to each prediction date, similar to prior work^9,13^.

For each individual, prediction instances were sampled at successive 6-month landmarks, starting from the date of the first available record across all data domains. At each landmark, the observation window included all historical data recorded up to that time point, which were used for predictor extraction. Outcomes were then assessed over forward-looking prediction horizons of 1, 2, and 3 years. This approach allowed each individual to contribute multiple time-stamped prediction instances as their social care record accumulated over time.

Follow-up for each individual continued until death or the administrative censoring date, 1 May 2024, whichever occurred first. For care-plan initiation and hospital-admission outcomes, instances in which an individual died within the prediction horizon before experiencing the non-mortality outcome were treated as right-censored rather than labelled as negatives. The implications of this choice are considered in the Discussion.

Prediction instances were excluded if, at the prediction date, an active care plan was already present for the care-plan prediction task, or an ongoing hospital admission was in progress for the hospital-admission prediction task. Predictors were derived independently for each prediction instance, using only information available prior to or on the corresponding prediction date. Outcome information after the prediction date was used only for label construction, ensuring that no future information beyond the prediction date was used during predictor extraction.

After applying instance-level eligibility criteria, 27,590 individuals contributed at least one valid prediction instance, resulting in 291,392 care-plan, 379,118 hospital-admission, and 389,148 mortality prediction instances (Fig. 1b). In total, 198 predictors were constructed across all seven domains, including binary indicators, event durations, and free-text-derived health-condition embeddings. Detailed predictor definitions and preprocessing steps are provided in Supplementary Section S1.

### Predictors

Our analysis leverages a diverse array of features derived from the rich longitudinal social care records. These predictors capture various dimensions of patient care, ranging from basic demographics to detailed clinical and service utilisation metrics. In brief, the key predictors extracted before the prediction date include:

Demographic Features: Age (in years at baseline) and gender (encoded as Male = 0, Female = 1, and Unknown = 0.5).

Care Assessments: Thirteen binary indicators capturing whether specific care assessments occurred during the observation period. These assessments include, for example, the OT (Occupational Therapy) Specialist Assessment SAP, Care Act Assessment, Occupational Therapy Assessment, OT Seating and Posture Assessment, OT Moving and Handling Assessment, Hearing Impairment Evaluation, Visual Sensory Impairment Specialist Assessment SAP, Hearing Sensory Impairment Specialist Assessment SAP, Carers Assessment, Visual Impairment Evaluation, Occupational Therapy Moving & Handling and Seating & Posture Assessment, Occupational Therapy Re-assessment, and Dual Sensory Loss Assessment.

Long-Term Care Plans: A single feature representing the total duration (in months) of long-term care plans documented in the historical records. This is simply accumulated if there are multiple plans in the previous records.

OT Equipment and Reablement: Two features quantifying the frequency of OT equipment assessments (specifically, Moving and Handling Assessment and Seating and Posture Assessment) and one binary indicator denoting whether a “home-first" reablement form was completed.

Service utilisation: 36 features summarizing patient engagements across 12 service groups (e.g., Home Support - OCC, Day Services, Care Home Placement, Transport, Direct Payments - OCC, Shared Lives, Other Community Support, Health Funded Services, Extra Care Housing, Supported Living, Direct Payments - Carers, and SEN Placement). For each group, we computed the total number of engagements, the average duration (in months), and the standard deviation of engagement durations.

Health Conditions: 70 features derived from 20 predefined health conditions (such as Stroke, Chronic Obstructive Pulmonary Disease (COPD), Dementia, Parkinson’s, etc.). For each condition, we extracted a binary indicator of occurrence during the observation period along with the total duration. In addition, free-text entries for “Other Condition” were processed using the BiomedBERT (Biomedical Bidirectional Encoder Representations from Transformers) model^1^. The resulting 768-dimensional embeddings were then reduced to 10 principal components using Principal Component Analysis (PCA, based on a training subset each time) to maintain computational efficiency while preserving essential information.

Hospital Admissions: 3 features summarising historic hospital admissions: the total number of admissions, the average length of hospital stays (in days), and the standard deviation of these durations.

For a complete overview of the feature extraction process, including detailed variable definitions and extraction procedures, please refer to Supplementary Section S1.

### Predictive modelling procedure

Using the constructed prediction instances, we developed predictive models to estimate the risk of care-plan initiation, hospital admission, and mortality over fixed prediction horizons of 1, 2, and 3 years. For each outcome and horizon, the primary modelling task was formulated as a binary classification problem, where models estimated the probability that the outcome would occur within the specified future window.

We evaluated three commonly used supervised learning approaches with differing model capacities: logistic regression (LR), random forest (RF), and gradient-boosted decision trees implemented using XGBoost. These models were selected to provide a comparative assessment across linear, ensemble-based, and nonlinear boosting methods under the same repeated prediction framework.

All models were trained using subject-wise five-fold cross-validation, repeated across 10 random seeds to account for variability in fold assignment. Within each run, all prediction instances from a given individual were assigned exclusively to either the training or test set, preventing both subject-level and temporal information leakage. Model outputs were post-hoc calibrated using sigmoid scaling fitted on the training data and applied to held-out test predictions.

### Model evaluation

We assessed model performance on held-out test folds using metrics aligned with the fixed-horizon prediction task. For binary classification, we reported the area under the receiver operating characteristic curve (AUROC) and the area under the precision–recall curve (AUPRC) to evaluate discrimination, particularly under class imbalance. In addition, we reported accuracy and F1 score to summarise overall classification performance and the balance between precision and recall. Model calibration was assessed using calibration curves and the Brier score, which quantifies the mean squared error between predicted probabilities and observed outcomes.

All metrics were computed on held-out test folds and averaged across repeated cross-validation runs.

Predicted risks from the fixed-horizon models were subsequently used for post-hoc analyses to examine whether model-derived risk scores reflected meaningful longitudinal outcome patterns. These analyses were descriptive and were not used for model training, hyperparameter tuning, calibration, threshold selection, or model optimisation. Eligible instances for these post-hoc analyses were defined using baseline task eligibility at the prediction date, for example excluding individuals with an active care plan for the care-plan task or an ongoing hospital admission for the hospital-admission task, but not excluding instances solely because they were later censored or experienced death before the target endpoint.

For population-level risk stratification, individuals were grouped according to their calibrated 1-year predicted risks across care-plan initiation, hospital admission, and mortality, and Kaplan–Meier curves were used to compare subsequent all-cause survival across joint risk phenotypes. For the two non-mortality endpoints, care-plan initiation and hospital admission, we additionally performed competing-risk sensitivity analyses in which death before the target endpoint was treated as a competing event. We estimated Aalen–Johansen cumulative incidence functions by predicted-risk group and fitted cause-specific Cox models using the calibrated fixed-horizon risk score as the exposure.

## Supplementary information


Supplementary Information


## Data Availability

This work was approved by Oxfordshire County Council’s Data Governance Board and conducted under a data-sharing agreement between Oxfordshire County Council and the University of Oxford. The datasets generated and/or analyzed during the current study are not publicly available due to governance restrictions but are available from the corresponding author on reasonable request, which is also subject to approval by Oxfordshire County Council.

## Footnotes

1 https://huggingface.co/microsoft/BiomedNLP-BiomedBERT-base-uncased-abstract

## Code availability

The data analysis script, with synthetic examples, will be made available at https://github.com/guxiao0822/socialcareupon acceptance.
