# Supplementary material for: Anticipating health and care trajectories from routinely collected social care records
Source: Npj Health Syst. 2026 Aug 29;3:78. doi: 10.1038/s44401-026-00139-3 (PMC13526014; doi:10.1038/s44401-026-00139-3)
Supplement: Supplementary file 1 [file 44401_2026_139_MOESM1_ESM.pdf]

Supplementary Materials

S1 Supplementary Data Details

S1.1 Raw Data Description

The anonymised social care dataset provided by Oxfordshire County Council comprised multiple relational tables, each linked through a unique identifier (*Dummy LAS ID*). The raw data covered 30,913 individuals and included detailed records on demographics, assessments, service use, health conditions, and hospital admissions.

Person Demographics. This table contained one record per individual, including *year of death*, *ethnicity*, *gender*, *nationality*, *religion*, *language*, *age or age at death*, and *current funding status*. Typical entries included gender (`Male`, `Female`, `Not Known`) and ethnicity (`White - British`, `Chinese`, `Not Known`). The median age was 73 years (IQR 57–82).

Care Assessment. Each record represented a completed or cancelled care assessment and included the *completion date* and *form type*. Assessment types included `Care Act Assessment`, `Occupational Therapy (OT) Specialist Assessment SAP`, `Carers Assessment`, and `Sensory Impairment Specialist Assessments`. Some individuals had multiple assessments over time.

Care Plan – LT. Long-term care plans recorded the *effective from* and *effective to* dates, *plan status* (e.g., `Active`, `Completed`, `Superseded`), and *plan type* (`Adults`). Each entry corresponded to a formal period of commissioned care.

OT Equipment. This table contained occupational therapy-related equipment and assessment records, listing the *form type* (e.g., `OT Moving and Handling Assessment`, `OT Seating and Posture Assessment`), *completion date*, and responses such as `Equipment only`. These records reflect the provision of assistive equipment to support independent living.

Reablement. Reablement records captured short-term rehabilitation or discharge support activity, including *form type* (`Home First Assessment Form`, `Home First Referral Form`) and *completion date*. Each entry denotes a distinct reablement episode intended to restore functional independence after hospital discharge.

Services. The services table documented all social care services commissioned for individuals, including the *service group class*, *start date*, and *end date*. Common service categories were `Home Support - OCC`, `Day Services`, `Care Home Placement`, `Direct Payments`, `Shared Lives`, `Health-Funded Services`, and `Supported Living`. Each record corresponds to one episode of service provision.

Hospital Admissions. Hospital admission data included *admission date*, *expected discharge date*, *actual discharge date*, and *hospital name*. Hospitals appearing in the records included John Radcliffe Hospital, Horton Hospital, Witney Community Hospital, and Nuffield Orthopaedic Centre. Each record represents a single inpatient episode.

Health Conditions. This table recorded diagnosed or self-reported conditions, with *start and end dates*, *condition type*, *condition description*, and *free-text "Other Condition"*. Common condition types were `Long-Term Health Condition - Physical`, `Mental Health Condition`, and `Long-Term Health Condition - Neurological`. Frequent entries included stroke, dementia, Parkinson's disease, and osteoarthritis.

Together, these tables capture a detailed, longitudinal account of social care interactions and health status across Oxfordshire residents, forming a comprehensive foundation for population-level modelling of care needs and outcomes.

S1.2 Data Preprocessing and Feature Lists

Before analysis, all raw data tables were cleaned, harmonized, and linked through the anonymized identifier (*Dummy LAS ID*). Duplicate person identifiers were removed. Cohort-level exclusions were applied to individuals who were younger than 18 years (2,879) at their first usable record or subsequently who had demographic-only records (347), defined as having no usable dated non-demographic record across the linked service-use, care-assessment, care-plan, hospital-admission, health-condition, occupational-therapy, or reablement domains. This yielded the final analytical cohort of 27,590 adults.

Record alignment and date harmonization. All date fields (e.g., assessment, admission, service start / end, and condition onset / end) were converted to ISO 8601 format and coerced to consistent datetime types. For each record type, the earliest valid date per individual was identified to derive "first-event" markers such as *Care Assessment First Date*, *Care Plan First Date*, and *Hospital Admission First Date*. These were used to define each person's initial engagement with social care services and to establish longitudinal sequencing across tables.

Feature extraction. Predictors were aggregated within a dynamic observation window preceding each prediction date, as described in the main text (Figure 2). Feature groups are summarized in Table S2.

All numeric features were z-normalized and missing values were imputed using within-domain medians. Condition-related features were derived by parsing structured condition tables and applying rule-based logic to create event-start (*Condition Happens_**), event-end (*Condition Ends_**), and duration (*duration_of_**) summaries for each diagnostic category.

Label curation and censoring. Outcome labels were defined for three targets, care plan initiation, hospital admission, and mortality, within fixed prediction horizons (1-, 2-, 3-year). For care plan and hospital admission tasks, records overlapping

Table S1. Raw Data Schema of the Oxfordshire Adult Social Care Records. The anonymised dataset comprises 28,019 unique individuals and ~250,000 longitudinal records across all tables. All tables are linked through the anonymised identifier (*Dummy LAS ID*). For categorical variables with a limited number of options, representative *Unique Values* are shown; for numeric, free-text, or high-cardinality variables, illustrative *Example Values* are provided.

Table	Field Name	Data Type	Unique Values	Example Values
**Person Demographics**	Year of Death	Numeric (YYYY)	—	2017, 2021, NA
	Gender Description	Categorical	Male, Female, Not Known	—
	Ethnicity Description	Categorical	—	White – British, Chinese, Not Known
	Funding (current services)	Categorical	—	Council Funded, No current service
**Care Assessment**	Person Form Completion Date	Date	—	2019-03-11
	Person Form Type Description	Categorical	—	Care Act Assessment, Carers Assessment, OT Specialist Assessment SAP
**Care Plan – LT**	Care Plan Effective From Date	Date	—	2016-01-25
	Plan Status Description	Categorical	Active, Completed, Superseded	—
	Plan Type Description	Categorical	Adults	—
**OT Equipment**	Person Form Type Description	Categorical	OT Moving and Handling Assessment; OT Seating and Posture Assessment	—
	Person Forms Answer (Ans1)	Text	—	Equipment only
**Reablement**	Person Form Type Description	Categorical	Home First Assessment Form; Home First Referral Form	—
	Completion Date	Date	—	2023-06-15
**Services**	Service Group Class	Categorical	Home Support – OCC; Day Services; Care Home Placement; Direct Payments – OCC; Shared Lives; Supported Living; Health Funded Services; Extra Care Housing; Other Community Support; Direct Payments – Carers; SEN Placement; Transport	—
	Start Date / End Date	Date	—	2018-05-21 / 2019-03-11
**Hospital Admissions**	Admission Date / Discharge Date	Date	—	2023-08-14 / 2023-09-15
	Hospital Name	Categorical	—	John Radcliffe Hospital; Horton Hospital; Nuffield Orthopaedic Centre
**Health Conditions**	Condition Type Description	Categorical	Long-Term Health Condition – Physical; Mental Health Condition; Long-Term Health Condition – Neurological	—
	Condition Description	Categorical	—	Stroke; Dementia; Other Physical Health Condition; etc.
	Other Condition	Free Text	—	Anxiety; Osteoarthritis affecting hands

the prediction date (i.e., active episodes) were excluded to ensure prospective prediction. Cases ending before the prediction window were treated as right-censored.

S2 Supplementary Model Development Details

S2.1 Basic Classifier Settings

We compared three baseline machine learning models, logistic regression, random forest, and gradient-boosted trees (XGBoost), implemented in `scikit-learn` (v1.5) and `xgboost` (v2.0). Each model was wrapped in a preprocessing pipeline. Numeric predictors were imputed using a `SimpleImputer` with median strategy, and the free-text health-condition embedding was zero-imputed before PCA. Preprocessing steps were fitted exclusively on the training folds and then applied to the held-out folds to avoid information leakage.

Logistic Regression. We used an ℓ_2-penalized logistic regression model (`solver=lbfgs`, `max_iter=2000`) with class-weight balancing (`class_weight=balanced`) to correct for label imbalance. Continuous predictors were standardized within each fold. This model provides a well-calibrated linear baseline and interpretable coefficients.

Table S2. Predictors used for model development. Predictors were derived from routinely collected Oxfordshire Adult Social Care records. All predictors were constructed at landmark time points using only information available on or before the landmark date to ensure leakage-free prediction. For time-varying records, `count` denotes the number of recorded episodes prior to the landmark; `dur_sum_days` denotes the cumulative duration (in days) across episodes; `dur_mean_days` and `dur_std_days` denote the mean and standard deviation of episode duration; and `active` indicates whether the service, condition, or plan was ongoing at the landmark time point.

Domain	Predictors
**Demographics (2)**	Gender and age measured at the landmark time point: `gender`, `age`
**Service utilisation (60)**	For each community service category (Care Home Placement, Day Services, Direct Payments – Carers, Direct Payments – OCC, Extra Care Housing, Health Funded Services, Home Support – OCC, Other Community Support, SEN Placement, Shared Lives, Supported Living, Transport) recorded prior to the landmark: `svc_count__*`, `svc_dur_sum_days__*`, `svc_dur_mean_days__*`, `svc_dur_std_days__*`, `svc_active__*`
**Hospital admissions (5)**	Summary measures of all hospital admissions recorded prior to the landmark time point: `ha_count`, `ha_los_sum_days`, `ha_los_mean_days`, `ha_los_std_days`, `ha_active`
**Health conditions (100+10)**	For each recorded health condition (Acquired Brain Injury, Acquired Physical Injury, Asperger's Syndrome / High Functioning Autism, Autism (excluding Asperger's), Autism Spectrum Diagnosis, Cancer, Chronic Obstructive Pulmonary Disease, Dementia, HIV / AIDS, Hearing Impaired, Learning Disability, Motor Neurone Disease, Other Disability, Other Neurological Health Condition, Other Physical Health Condition, Other Sensory Impairment, Parkinson's, Stroke, Visually Impaired, Other Condition), based on records prior to the landmark: `hc_count__*`, `hc_dur_sum_days__*`, `hc_dur_mean_days__*`, `hc_dur_std_days__*`, `hc_active__*` Other health conditions obtained by averaging pretrained text embeddings of historical free-text "Other Condition" entries recorded prior to the landmark and subsequently reduced to 10 components: `hc_other_emb_1` – `hc_other_emb_10`
**Care assessments (13)**	Counts of each type of care assessment completed prior to the landmark (OT Specialist Assessment SAP, Care Act Assessment, Occupational Therapy Assessment, OT Seating and Posture Assessment, OT Moving and Handling Assessment, Hearing Impairment, Visual Sensory Impairment Specialist Assessment SAP, Hearing Sensory Impairment Specialist Assessment SAP, Carers Assessment, Visual Impairment, Occupational Therapy Moving & Handling and Seating & Posture Assessment, Occupational Therapy – Re-assessment, Dual Sensory Loss): `ca_count__*`
**Care plans – long term (5)**	Summary measures of long-term care plans recorded prior to the landmark: `cp_count`, `cp_dur_sum_days`, `cp_dur_mean_days`, `cp_dur_std_days`, `cp_active`
**OT equipment and reablement (3)**	Counts of occupational therapy equipment provision and reablement activity recorded prior to the landmark: `ot_count__moving_and_handling`, `ot_count__seating_and_posture`, `re_home_first_count`
**Total**	198 predictors

Random Forest. A random forest classifier (`n_estimators=500`, `max_depth=None`) with bootstrap sampling and balanced class weights (`class_weight=balanced_subsample`) was employed. This setting was selected after a compact development-stage check on the three 1-year prediction tasks, where we examined `n_estimators` $\in \{100, 500, 1000\}$ while keeping the remaining random forest settings fixed.

XGBoost. The gradient-boosted decision tree model was trained using `n_estimators=800`, learning rate = 0.03, maximum depth = 5, subsample ratio = 0.8, column subsample ratio = 0.8, and ℓ_2 regularization $\lambda = 1.0$. These settings were selected after a compact one-at-a-time development-stage check on the three 1-year prediction tasks, where we examined `n_estimators` $\in \{100, 800, 1000\}$ and learning rate $\in \{0.003, 0.03, 0.3\}$ around the reported configuration.

Cross-validation. All models were evaluated using five-fold *group* cross-validation (`GroupKFold`), with grouping by unique individual identifier to prevent data from the same person appearing in both training and testing folds. Model training and evaluation were repeated under ten random seeds (42,7,13,21,37,101,123,2023,2024,2025) to assess stability. For each seed, data were randomly permuted prior to fold generation.

Performance metrics. For each task and prediction horizon, models were evaluated by the area under the receiver operating characteristic curve (AUROC) and the area under the precision–recall curve (AUPRC). To quantify uncertainty, 95% confidence intervals were estimated using 500 bootstrap replicates resampled at the individual level.

Table S3. Quantitative performance of three outcomes across prediction tasks and horizons.

	Care Plan			Hospital Admission			Mortality		
Metric	1 year	2 years	3 years	1 year	2 years	3 years	1 year	2 years	3 years
Instances (N)	255,811	227,841	205,541	333,854	298,296	269,770	352,994	320,880	294,003
Positive instances (N)	21,216	34,622	45,313	19,369	33,505	44,911	17,585	32,143	44,609
Positive rate (%)	8.29	15.20	22.05	5.80	11.23	16.65	4.98	10.02	15.17
AUROC ↑	0.749 [0.748, 0.750]	0.738 [0.736, 0.739]	0.747 [0.746, 0.748]	0.795 [0.793, 0.796]	0.806 [0.805, 0.807]	0.821 [0.819, 0.823]	0.874 [0.874, 0.875]	0.883 [0.882, 0.884]	0.893 [0.892, 0.894]
AUPRC ↑	0.318 [0.316, 0.320]	0.398 [0.396, 0.401]	0.510 [0.509, 0.512]	0.202 [0.199, 0.205]	0.375 [0.373, 0.376]	0.535 [0.529, 0.540]	0.280 [0.278, 0.283]	0.477 [0.474, 0.479]	0.638 [0.634, 0.642]
Accuracy ↑	0.921 [0.921, 0.922]	0.851 [0.850, 0.852]	0.797 [0.797, 0.798]	0.939 [0.938, 0.939]	0.887 [0.887, 0.888]	0.853 [0.852, 0.854]	0.945 [0.944, 0.945]	0.906 [0.905, 0.907]	0.884 [0.883, 0.884]
Brier score ↓	0.068 [0.067, 0.068]	0.116 [0.115, 0.116]	0.148 [0.147, 0.148]	0.052 [0.051, 0.052]	0.086 [0.086, 0.087]	0.108 [0.108, 0.109]	0.043 [0.043, 0.043]	0.069 [0.069, 0.070]	0.085 [0.084, 0.085]

Table S4. Death-before-target non-case sensitivity analysis for fixed-horizon CP/HA classifiers. Primary labelled rows reproduce the XGBoost results reported in Table S3. Sensitivity rows use the same ten seeds and add held-out death-before-target rows as non-cases, while keeping the original positive-event labels unchanged.

Outcome	Analysis	Horizon	Instances	Events	Death non-cases	Event rate	AUROC	AUPRC	Brier
Care plan	Primary labelled	1 year	255,811	21,216	–	0.083	0.749 [0.748, 0.750]	0.318 [0.316, 0.320]	0.068 [0.067, 0.068]
Care plan	Death retained as non-case	1 year	264,108	21,216	8,297	0.080	0.740 [0.740, 0.741]	0.295 [0.294, 0.295]	0.068 [0.068, 0.068]
Care plan	Primary labelled	2 years	227,841	34,622	–	0.152	0.738 [0.736, 0.739]	0.398 [0.396, 0.401]	0.116 [0.115, 0.116]
Care plan	Death retained as non-case	2 years	240,866	34,622	13,025	0.144	0.718 [0.718, 0.719]	0.349 [0.348, 0.349]	0.119 [0.119, 0.119]
Care plan	Primary labelled	3 years	205,541	45,313	–	0.220	0.747 [0.746, 0.748]	0.510 [0.509, 0.512]	0.148 [0.147, 0.148]
Care plan	Death retained as non-case	3 years	222,133	45,313	16,592	0.204	0.714 [0.714, 0.714]	0.408 [0.407, 0.408]	0.161 [0.161, 0.161]
Hospital admission	Primary labelled	1 year	333,854	19,369	–	0.058	0.795 [0.793, 0.796]	0.202 [0.199, 0.205]	0.052 [0.051, 0.052]
Hospital admission	Death retained as non-case	1 year	346,998	19,369	13,144	0.056	0.785 [0.785, 0.785]	0.179 [0.179, 0.180]	0.051 [0.051, 0.051]
Hospital admission	Primary labelled	2 years	298,296	33,505	–	0.112	0.806 [0.805, 0.807]	0.375 [0.373, 0.376]	0.086 [0.086, 0.087]
Hospital admission	Death retained as non-case	2 years	320,048	33,505	21,752	0.105	0.782 [0.782, 0.782]	0.298 [0.298, 0.299]	0.090 [0.090, 0.090]
Hospital admission	Primary labelled	3 years	269,770	44,911	–	0.166	0.821 [0.819, 0.823]	0.535 [0.529, 0.540]	0.108 [0.108, 0.109]
Hospital admission	Death retained as non-case	3 years	298,046	44,911	28,276	0.151	0.780 [0.779, 0.780]	0.378 [0.378, 0.379]	0.126 [0.126, 0.126]

S3 Additional Results

S3.1 Detailed quantitative results of fixed-horizon predictions

Additional quantitative results for the primary fixed-horizon prediction models across outcomes, horizons, and classifiers are provided in Table S3. These results correspond to the primary labelled binary tasks described in the Methods.

S3.2 Death-before-target non-case sensitivity analysis

As a supplementary sensitivity analysis for the two non-mortality endpoints, we retained the original fixed-horizon positive-event definitions and added held-out death-before-target rows back into the evaluation set as non-cases. In other words, the number of positive care-plan initiation and hospital-admission events was unchanged from the primary Stage 4 binary labels; only the evaluation denominator was expanded by adding instances in which death occurred before the target endpoint. To keep the comparison aligned with Table S3, the primary labelled rows below reproduce the XGBoost results reported in that table, while the sensitivity rows were summarized across the same ten random seeds used for the main fixed-horizon experiments. This analysis was intended to test whether the original calibrated fixed-horizon risk scores retained discriminatory signal when death-before-target rows were retained as non-cases. It was not intended to replace the primary binary classification analysis, and it does not constitute a formal competing-risk cumulative-incidence model.

Across both non-mortality endpoints, the held-out risk scores retained discriminatory signal after death-before-target rows were added as non-cases, although discrimination was lower than in the primary labelled evaluation. For care-plan initiation, AUROC changed from 0.749 to 0.740 at 1 year, from 0.738 to 0.718 at 2 years, and from 0.747 to 0.714 at 3 years. For hospital admission, AUROC changed from 0.795 to 0.785 at 1 year, from 0.806 to 0.782 at 2 years, and from 0.821 to 0.780 at 3 years. The positive-event counts were unchanged in all cases, while the event rates decreased after adding death-before-target non-cases. These results are consistent with the reviewer's concern that death handling affects fixed-horizon non-mortality endpoint interpretation, particularly at longer horizons. They also show that the main predictive signal was not removed by retaining death-before-target instances as non-cases in this multiseed sensitivity evaluation. The results should therefore be interpreted as supplementary sensitivity evidence only; the main competing-risk interpretation is based on the post-hoc Aalen–Johansen cumulative-incidence and cause-specific Cox analyses reported in the main text.

S3.3 Subgroup performance

We performed additional subgroup analyses to examine whether model performance and landmark contribution varied across baseline demographic groups. Subgroups were defined using demographic information from each individual's first social-care record, consistent with the cohort summary in Figure **??**d.

Across 27,590 individuals, each person contributed a median of 10 six-month landmark prediction instances (IQR 4–20; mean 14.1; range 1–153). Landmark contribution differed most clearly by baseline age group: individuals aged <50 years contributed a median of 17 landmarks (IQR 7–34), whereas those aged >70 years contributed a median of 8 landmarks (IQR

Table S5. Number of six-month landmark prediction instances contributed per person. Baseline demographic groups were defined from each individual's first social-care record.

Grouping	Group	People	Mean	SD	Median	IQR
Overall	All individuals	27,590	14.1	12.9	10	4–20
Age group	<50	4,838	21.9	18.4	17	7–34
Age group	50–60	2,745	17.7	13.7	15	6–27
Age group	60–70	3,821	16.7	12.5	14	6–26
Age group	>70	16,186	10.6	8.9	8	4–15
Sex	Male	11,361	13.1	13.0	9	4–18
Sex	Female	16,215	14.8	12.8	11	5–22
Sex	Unknown	14	6.6	4.6	6	2–12
Ethnicity	White	23,841	14.8	13.1	11	5–22
Ethnicity	Asian or Asian British	637	14.8	13.0	11	5–21
Ethnicity	Black or Black British	386	15.7	13.3	11	5–24
Ethnicity	Mixed	198	12.4	12.2	8	4–18
Ethnicity	Other ethnic groups	261	10.0	10.1	5	3–13
Ethnicity	Not stated / unknown	2,267	7.1	7.8	5	3–9

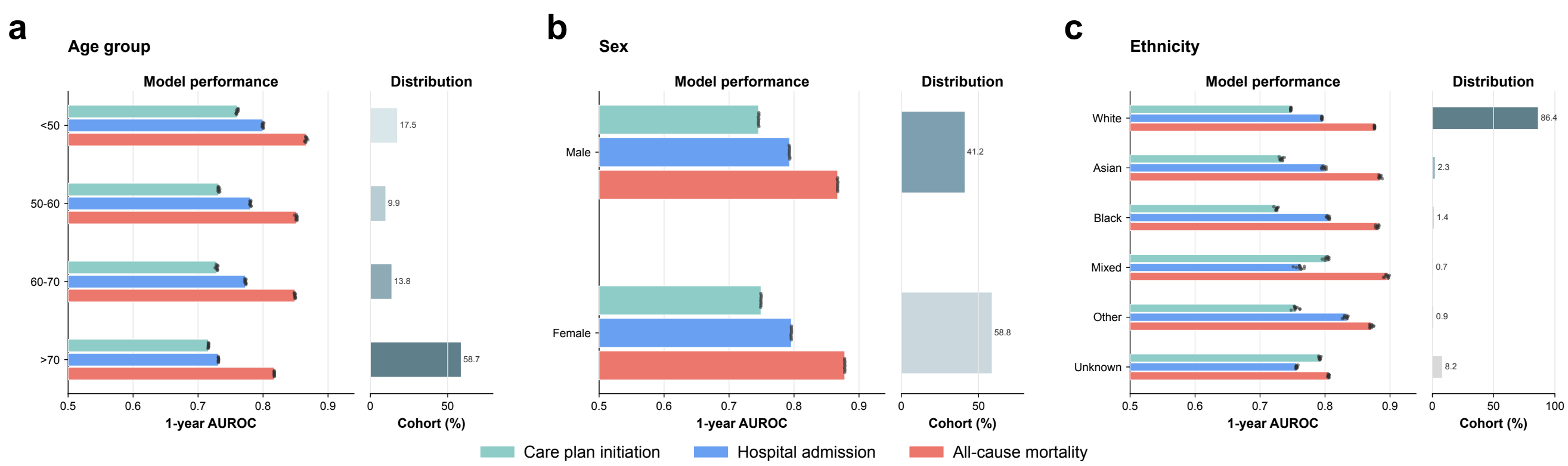

Figure S1. Subgroup performance of 1-year XGBoost models. Subgroups were defined using demographic information from each individual's first social-care record. For each subgroup, the left panel shows model discrimination, reported as mean AUROC across ten random seeds, with seed-level estimates overlaid as black points. The right panel shows the proportion of individuals in the cohort belonging to each subgroup. The unknown-sex group (14 individuals) was retained in descriptive cohort summaries but not shown here.

4–15). Differences by sex were smaller, with males contributing a median of 9 landmarks (IQR 4–18) and females 11 landmarks (IQR 5–22).

We also evaluated 1-year XGBoost model discrimination across baseline age group, sex, and ethnicity using the ten-seed cross-validation outputs. Performance was broadly similar by sex. By age group, AUROC was lower among individuals aged >70 years across all three outcomes, which may reflect greater clinical and care heterogeneity, higher baseline event rates, shorter observable follow-up, and reduced separability within this older subgroup. Ethnicity-based estimates should be interpreted cautiously because several groups had small sample sizes and fewer events.

All cross-validation was performed at the individual level, so landmarks from the same person were kept within the same fold. However, the repeated-landmark design does not equalise the number of prediction instances contributed by each individual. These subgroup analyses should therefore be interpreted as descriptive checks of performance heterogeneity, rather than as formal fairness or transportability analyses.

S3.4 Feature-selection sensitivity analysis

The primary models used an a priori predictor set derived from routinely available social-care domains, rather than outcome-driven feature selection. To assess whether the findings depended on retaining the full predictor set, we performed a post-hoc feature-selection sensitivity analysis for the 1-year XGBoost models.

Within each training fold, we first fitted the full 198-feature XGBoost model and ranked model-input features by XGBoost gain. We then refitted reduced XGBoost models using only the top 20, top 50, or top 100 ranked features from that training fold, and evaluated each reduced model on the held-out fold. Feature selection was therefore performed entirely within the training data for each fold, avoiding use of held-out outcome information during feature selection.

As shown in Supplementary Figure S2, the top-100 feature models retained near-identical discrimination to the full predictor model across all three outcomes. AUROC changed from 0.7486 to 0.7470 for care-plan initiation, from 0.7947 to 0.7947 for hospital admission, and from 0.8743 to 0.8742 for all-cause mortality. Models restricted to the top 20 features showed larger performance reductions, particularly for care-plan initiation. These findings suggest that a smaller subset of variables captures much of the predictive signal, but that the broader a priori predictor set remains useful, especially for the more heterogeneous care-plan endpoint.

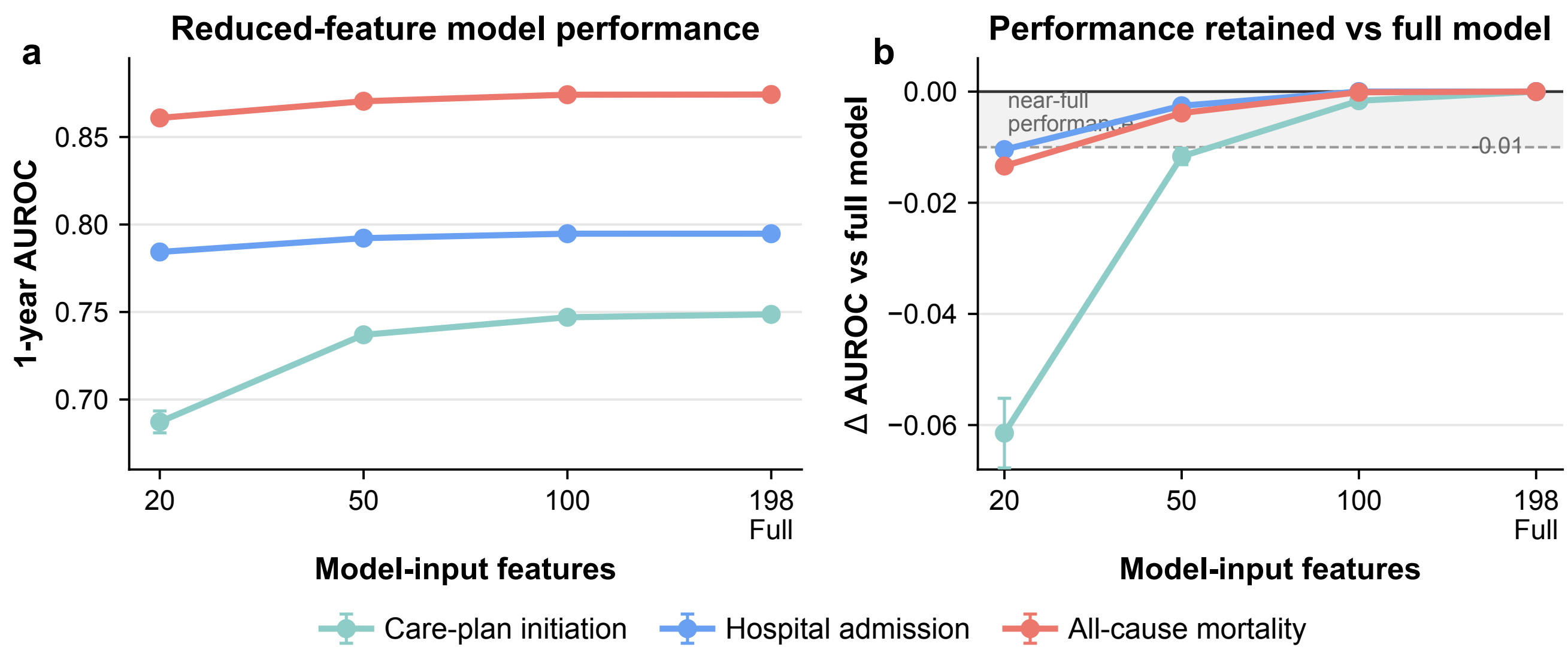

Figure S2. Fold-internal feature-selection sensitivity analysis. For each 1-year XGBoost task, model-input features were ranked within each training fold using XGBoost gain from the full 198-feature model. Reduced models were then refitted using the top 20, top 50, or top 100 ranked features and evaluated on the held-out fold. **a.** AUROC of reduced-feature and full models. **b.** Difference in AUROC relative to the full 198-feature model; the shaded region indicates AUROC within 0.01 of the full model. Points show mean performance across ten random seeds, and error bars show the standard deviation across seeds.

Section/Topic	Item	Development / evaluation[1]	Checklist item	Reported on page
**TITLE**				
Title	1	D;E	Identify the study as developing or evaluating the performance of a multivariable prediction model, the target population, and the outcome to be predicted	1
**ABSTRACT**				
Abstract	2	D;E	See TRIPOD+AI for Abstracts checklist	1
**INTRODUCTION**				
Background	3a	D;E	Explain the healthcare context (including whether diagnostic or prognostic) and rationale for developing or evaluating the prediction model, including references to existing models	1,9
	3b	D;E	Describe the target population and the intended purpose of the prediction model in the context of the care pathway, including its intended users (e.g., healthcare professionals, patients, public)	1,3,9-10
	3c	D;E	Describe any known health inequalities between sociodemographic groups	9-10,18
Objectives	4	D;E	Specify the study objectives, including whether the study describes the development or validation of a prediction model (or both)	1,3
**METHODS**				
Data	5a	D;E	Describe the sources of data separately for the development and evaluation datasets (e.g., randomised trial, cohort, routine care or registry data), the rationale for using these data, and representativeness of the data	3,10,14
	5b	D;E	Specify the dates of the collected participant data, including start and end of participant accrual; and, if applicable, end of follow-up	3,10,14
Participants	6a	D;E	Specify key elements of the study setting (e.g., primary care, secondary care, general population) including the number and location of centres	3,10
	6b	D;E	Describe the eligibility criteria for study participants	3,10,14
	6c	D;E	Give details of any treatments received, and how they were handled during model development or evaluation, if relevant	10-11, 14,16
Data preparation	7	D;E	Describe any data pre-processing and quality checking, including whether this was similar across relevant sociodemographic groups	14-16
Outcome	8a	D;E	Clearly define the outcome that is being predicted and the time horizon, including how and when assessed, the rationale for choosing this outcome, and whether the method of outcome assessment is consistent across sociodemographic groups	10,17
	8b	D;E	If outcome assessment requires subjective interpretation, describe the qualifications and demographic characteristics of the outcome assessors	na
	8c	D;E	Report any actions to blind assessment of the outcome to be predicted	na
Predictors	9a	D	Describe the choice of initial predictors (e.g., literature, previous models, all available predictors) and any pre-selection of predictors before model building	11, 16, 18-19
	9b	D;E	Clearly define all predictors, including how and when they were measured (and any actions to blind assessment of predictors for the outcome and other predictors)	11,14,16
	9c	D;E	If predictor measurement requires subjective interpretation, describe the qualifications and demographic characteristics of the predictor assessors	na
Sample size	10	D;E	Explain how the study size was arrived at (separately for development and evaluation), and justify that the study size was sufficient to answer the research question. Include details of any sample size calculation	3,10-11,17
Missing data	11	D;E	Describe how missing data were handled. Provide reasons for omitting any data	14-15,17
Analytical methods	12a	D	Describe how the data were used (e.g., for development and evaluation of model performance) in the analysis, including whether the data were partitioned, considering any sample size requirements	10-12,16
	12b	D	Depending on the type of model, describe how predictors were handled in the analyses (functional form, rescaling, transformation, or any standardisation).	11,14-16
	12c	D	Specify the type of model, rationale[2], all model-building steps, including any hyperparameter tuning, and method for internal validation	11,15-16
	12d	D;E	Describe if and how any heterogeneity in estimates of model parameter values and model performance was handled and quantified across clusters (e.g., hospitals, countries). See TRIPOD-Cluster for additional considerations[3]	9-10,18
	12e	D;E	Specify all measures and plots used (and their rationale) to evaluate model performance (e.g., discrimination, calibration, clinical utility) and, if relevant, to compare multiple models	3-5, 11-12, 16-17
	12f	E	Describe any model updating (e.g., recalibration) arising from the model evaluation, either overall or for particular sociodemographic groups or settings	na
	12g	E	For model evaluation, describe how the model predictions were calculated (e.g., formula, code, object, application programming interface)	11-12,16
Class imbalance	13	D;E	If class imbalance methods were used, state why and how this was done, and any subsequent methods to recalibrate the model or the model predictions	11,15-16
Fairness	14	D;E	Describe any approaches that were used to address model fairness and their rationale	9-10,18
Model output	15	D	Specify the output of the prediction model (e.g., probabilities, classification). Provide details and rationale for any classification and how the thresholds were identified	8,12

[1] D=items relevant only to the development of a prediction model; E=items relating solely to the evaluation of a prediction model; D;E=items applicable to both the development and evaluation of a prediction model
[2] Separately for all model building approaches.
[3] TRIPOD-Cluster is a checklist of reporting recommendations for studies developing or validating models that explicitly account for clustering or explore heterogeneity in model performance (eg, at different hospitals or centres). Debray et al, BMJ 2023; 380: e071018 [DOI: 10.1136/bmj-2022-071018]

Training versus evaluation	16	D;E	Identify any differences between the development and evaluation data in healthcare setting, eligibility criteria, outcome, and predictors	11-12,16
Ethical approval	17	D;E	Name the institutional research board or ethics committee that approved the study and describe the participant-informed consent or the ethics committee waiver of informed consent	3,10
**OPEN SCIENCE**				
Funding	18a	D;E	Give the source of funding and the role of the funders for the present study	12
Conflicts of interest	18b	D;E	Declare any conflicts of interest and financial disclosures for all authors	12
Protocol	18c	D;E	Indicate where the study protocol can be accessed or state that a protocol was not prepared	na
Registration	18d	D;E	Provide registration information for the study, including register name and registration number, or state that the study was not registered	na
Data sharing	18e	D;E	Provide details of the availability of the study data	12
Code sharing	18f	D;E	Provide details of the availability of the analytical code[4]	12
**PATIENT & PUBLIC INVOLVEMENT**				
Patient & Public Involvement	19	D;E	Provide details of any patient and public involvement during the design, conduct, reporting, interpretation, or dissemination of the study or state no involvement.	na
**RESULTS**				
Participants	20a	D;E	Describe the flow of participants through the study, including the number of participants with and without the outcome and, if applicable, a summary of the follow-up time. A diagram may be helpful.	3,10-11,17
	20b	D;E	Report the characteristics overall and, where applicable, for each data source or setting, including the key dates, key predictors (including demographics), treatments received, sample size, number of outcome events, follow-up time, and amount of missing data. A table may be helpful. Report any differences across key demographic groups.	3,14,17-18
	20c	E	For model evaluation, show a comparison with the development data of the distribution of important predictors (demographics, predictors, and outcome).	na
Model development	21	D;E	Specify the number of participants and outcome events in each analysis (e.g., for model development, hyperparameter tuning, model evaluation)	17
Model specification	22	D	Provide details of the full prediction model (e.g., formula, code, object, application programming interface) to allow predictions in new individuals and to enable third-party evaluation and implementation, including any restrictions to access or re-use (e.g., freely available, proprietary)[5]	11,15-16
Model performance	23a	D;E	Report model performance estimates with confidence intervals, including for any key subgroups (e.g., sociodemographic). Consider plots to aid presentation.	5,17-18
	23b	D;E	If examined, report results of any heterogeneity in model performance across clusters. See TRIPOD Cluster for additional details[3].	18
Model updating	24	E	Report the results from any model updating, including the updated model and subsequent performance	na
**DISCUSSION**				
Interpretation	25	D;E	Give an overall interpretation of the main results, including issues of fairness in the context of the objectives and previous studies	8-10
Limitations	26	D;E	Discuss any limitations of the study (such as a non-representative sample, sample size, overfitting, missing data) and their effects on any biases, statistical uncertainty, and generalizability	8-10
Usability of the model in the context of current care	27a	D	Describe how poor quality or unavailable input data (e.g., predictor values) should be assessed and handled when implementing the prediction model	10,12
	27b	D	Specify whether users will be required to interact in the handling of the input data or use of the model, and what level of expertise is required of users	10
	27c	D;E	Discuss any next steps for future research, with a specific view to applicability and generalizability of the model	10

From: Collins GS, Moons KGM, Dhiman P, et al. *BMJ* 2024;385:e078378. doi:10.1136/bmj-2023-078378

[4] This relates to the analysis code, for example, any data cleaning, feature engineering, model building, evaluation.
[5] This relates to the code to implement the model to get estimates of risk for a new individual.
